# CD58 reshapes the immunosuppressive microenvironment in gliomas through PD-L1 upregulation

**DOI:** 10.3389/fonc.2025.1658467

**Published:** 2025-12-08

**Authors:** Siqi Gou, Pengfei Zhao, Lei Zong, Daihua Yu, Wei Liu, Weichen Zuo, Juanhong Wang, Wei Wei

**Affiliations:** 1 Department of Pathology, Xi’an No. 3 Hospital, The Affiliated Hospital of Northwest University, Xi’an, Shaanxi, China; 2 Department of Neurosurgery, Affiliated Hospital of Hebei University, Baoding, Hebei, China; 3 Department of Critical Care Medicine, Xi’an No. 3 Hospital, The Affiliated Hospital of Northwest University, Xi’an, Shaanxi, China

**Keywords:** CD58, gliomas, microenvironment, PD-L1, chemokine, ICAM-1

## Abstract

**Background:**

Cluster of Differentiation 58 (CD58), a critical immune regulator, is implicated in tumor immune evasion, yet its role in remodeling the immunosuppressive microenvironment of gliomas and regulating programmed death-ligand 1 (PD-L1) remains unclear. This study aimed to elucidate the clinical and mechanistic significance of CD58 in gliomas.

**Methods:**

This study integrated bioinformatics analysis with *in vitro* validation. First, CD58 expression patterns, immunorelevance, prognostic significance, and clinical associations were evaluated using transcriptome data from TCGA (33 cancers, n=10,535) and CGGA (glioma, n=1,018). The immunomodulatory role of CD58 across tumors was assessed by comparing immune checkpoint molecules, chemokines, chemokine receptors, and immunoregulatory factors. ESTIMATE and CIBERSORT algorithms characterized the tumor immune microenvironment. CD58-related signaling pathways were identified through functional enrichment analysis (GO, KEGG, GSEA). *In vitro*, CD58 was knocked down in U87MG and LN229 glioma cells. Functional assays and protein expression validation were performed. Key cytokine levels (CCL5, CXCL9, CXCL10) secreted into conditioned media were quantified by ELISA to determine factors involved in CD58-mediated immune microenvironment remodeling.

**Results:**

In high-grade gliomas, the expression of CD58 was significantly increased, and it showed statistical differences in relation to clinical pathological indicators. Pan-cancer analysis revealed that CD58 was associated with key immune regulatory factors, including PD-L1, chemokines (CCL5, CXCL9, CXCL10). *In vitro* experiments demonstrated that knockdown of CD58 in glioma cells significantly inhibited proliferation, migration, and invasion, while enhancing the adhesion of T cells to glioma cells. Moreover, silencing of CD58 reduced the expression of PD-L1 and Vimentin, upregulated ICAM-1, and promoted the secretion of chemokines CCL5, CXCL9, and CXCL10.

**Conclusions:**

CD58 drives glioma progression by upregulating PD-L1 and reshaping the tumor microenvironment toward immunosuppression. It serves as an independent prognostic biomarker and a potential therapeutic target. Targeting the CD58-PD-L1 axis may enhance immunotherapy efficacy in gliomas.

## 1 Introduction

Glioma is the most common primary malignant tumor in the central nervous system (1). Despite advancements in multimodal therapies, the prognosis for glioblastoma patients remains poor, with a median survival of around 9 months and a 5-year survival rate below 10% (2, 3). While immune checkpoint inhibitors (ICIs) targeting the programmed death-1 (PD-1)/PD-L1 axis have transformed the treatment of various solid tumors, their effectiveness in gliomas is limited (4–6).This therapeutic resistance originates from the unique immunosuppressive tumor microenvironment (TME) of gliomas, primarily characterized by immune cell infiltration including tumor-associated macrophages (TAMs), regulatory T cells (Tregs), and myeloid-derived suppressor cells (MDSCs). These cells suppress effector T cell activity by secreting immunosuppressive factors such as IL-10 and TGF-β, thereby enhancing immune evasion. They also inhibit T cell and NK cell functions to promote tumor immune tolerance. Additionally, the blood-brain barrier restricts immune cell infiltration and therapeutic drug delivery. Consequently, gliomas are often referred to as “cold tumors” (5, 7–9).

CD58, also known as lymphocyte function-associated antigen-3 (LFA-3), is a costimulatory molecule primarily found on antigen-presenting cells (APCs) that plays a crucial role in T cell activation by interacting with the Cluster of Differentiation 2 (CD2) receptor on T cells (10, 11). Studies have demonstrated that CD58 expression in tumor cells influences tumor sensitivity to cytotoxic tumor-infiltrating lymphocytes (TILs) in a T cell receptor (TCR)-dependent manner (10, 12). The presence of an intact CD58-CD2 axis is crucial for effective TIL-mediated tumor cell lysis, and its disruption promotes immune evasion (11). Interestingly, CD58 deficiency has been associated with increased PD-L1 expression, suggesting a potential compensatory immunosuppressive mechanism (11). However, the reciprocal regulation between CD58 and PD-L1, and its functional implications in glioma immune modulation, have not been investigated.

This study introduces a novel hypothesis centered on the CD58-PD-L1 axis to address two primary inquiries: (1) the specific overexpression of CD58 in glioma and its correlation with the invasive phenotype and unfavorable prognosis, and (2) the regulatory role of CD58 in PD-L1 expression and its influence on the immunosuppressive tumor microenvironment. Through the integration of multi-omics data from TCGA and CGGA, along with functional validation in glioma cell lines U87MG and LN229, we systematically explored the mechanism underlying CD58’s function in glioma. These findings not only bridge existing knowledge gaps concerning the involvement of CD58 in neuro-oncology but also establish a theoretical foundation for the design of precise immunotherapeutic approaches targeting the CD58-PD-L1 axis.

## 2 Materials and methods

### 2.1 Data preparation for CD58 gene expression

Transcriptomic profiles, somatic mutation data, phenotypic information, and clinical records were obtained from two public databases: (1) the Sangerbox database (http://vip.sangerbox.com/login.html), which encompasses 34 human cancer types, and (2) TCGA (https://portal.gdc.cancer.gov/), covering 33 cancer types. Additionally, RNA sequencing data and clinical annotations for two independent glioma cohorts (n=325 and n=693) were retrieved from the CGGA (http://www.cgga.org.cn). Integrative pan-cancer analyses and visualization of TCGA multi-omics data were performed using R packages (version 4.2.1). The source code and prebuilt analytical pipelines are publicly accessible on GitHub (https://github.com/).

### 2.2 Correlation of CD58 gene expression with clinical features of gliomas

Glioma patients from the CGGA_325 and CGGA_693 cohorts were stratified into distinct subgroups based on age, gender, WHO grade, histological subtype, progression-free survival (PRS) classification, 1p/19q codeletion status, IDH mutation status, and chemotherapy history. CD58 mRNA expression levels were compared across these subgroups to evaluate associations with key clinicopathological characteristics of gliomas. Statistical significance was determined using the Kruskal-Wallis test or Mann-Whitney U test, as appropriate for data distribution.

### 2.3 Survival prognosis analysis

Kaplan-Meier survival analyses were performed using data from the CGGA_325 and CGGA_693 cohorts to evaluate the association between CD58 expression and overall survival (OS) in glioma patients. Receiver Operating Characteristic (ROC) curves were generated using the “timeROC” R package (version 4.2.1) to assess the predictive accuracy of CD58 for survival outcomes. Multivariate Cox proportional hazards regression analysis was performed to assess the independent prognostic value of CD58 expression. The model was adjusted for established clinical and molecular confounders, including age, WHO grade, IDH mutation status, and 1p/19q codeletion status. Additionally, multivariate Cox proportional hazards regression analysis and nomogram construction were conducted on the CGGA datasets using the “survival” R package (version 4.2.1) to quantify independent prognostic factors.

### 2.4 Gene enrichment analysis related to CD58

To identify biological pathways linked to CD58, Pearson correlation coefficients were calculated using the CGGA_325 and CGGA_693 datasets to extract genes co-expressed with CD58. The top 100 genes (50 most positively correlated and 50 most negatively correlated) were selected for functional annotation. Gene Ontology (GO) and Kyoto Encyclopedia of Genes and Genomes (KEGG) pathway analyses were performed using the Database for Annotation, Visualization and Integrated Discovery (DAVID, version 2021) with the Knowledgebase updated to v2023q4. Gene Set Enrichment Analysis (GSEA) was further conducted to explore CD58-related biological processes and signaling pathways. Significance thresholds were set at a false discovery rate (FDR) <0.05 and |normalized enrichment score (NES)| >1.5.

### 2.5 Patient samples

A total of 50 GBM tissue specimens were obtained from the Xi’an No. 3 Hospital, the Affiliated Hospital of Northwest University. All tumor resection specimens were collected from patients who had not been treated by chemo or radiotherapy prior to tumor surgery. A diagnosis of GBM in these samples was confirmed by trained pathologists. Informed consent was obtained from the patients, and the use of the tissue samples was approved by the Institutional Research Committee.

### 2.6 Immunohistochemistry

Immunohistochemical staining was performed to validate the expression of CD58 and PD-L1 in GBM tissues. This experiment was performed following standard procedures. The primary antibodies CD58 (1:500, Proteintech, Wuhan, China), PD-L1 (1:1000, Proteintech, Wuhan, China)were used. The percentage of the staining cells (P) was scored as fol lows: 0 (none), 1 (< 25% positive cells), 2 (25%–50% positive cells), 3 (> 50% positive cells). Staining intensity (I) was graded as follows: 0 (negative), 1 (weak yellow), 2 (medium yellow), 3 (deep yellow). Samples were evaluated for both factors, i.e. P multiplied by I. The samples with scoring > 3 were considered as positive.

### 2.7 Cell culture

The following human glioma cell lines were utilized in this study: U87MG, LN229, U251, and U373MG. The human embryonic kidney cell line 293T was included as a control. All cell lines were obtained from the Peking Union Basic Medical Cell Center (Beijing, China). Cells were cultured in Dulbecco’s Modified Eagle Medium (DMEM; KeyGEN BioTECH, Nanjing, China) supplemented with 10% fetal bovine serum (FBS; Gibco, Thermo Fisher Scientific, USA) and 1% penicillin-streptomycin (P/S; Gibco, Thermo Fisher Scientific, USA). Cultures were maintained at 37°C in a humidified atmosphere containing 5% carbon dioxide (CO_2_).

### 2.8 Plasmids

Upon reaching 90% confluence, U87MG and LN229 cells were transfected with either an empty plasmid (CSHCTR001-LVRH1MH; GeneCopoeia, Rockville, MD, USA) or a CD58 knockdown plasmid (EX-shLv201-CD58, Catalog #: HSH151222; GeneCopoeia). using the Lenti-Pac™ HIV Lentivirus Packaging System (GeneCopoeia). Plasmid DNA was purified using an endotoxin-free plasmid extraction kit (GeneJET Plasmid Maxiprep Kit; Thermo Fisher Scientific, Waltham, MA, USA). Lentiviral particles were generated by co-transfecting 293T cells with the packaging plasmids (pMD2.G and psPAX2) using polyethylenimine (PEI; Polysciences, Warrington, PA, USA). Viral supernatants were harvested 48 hours post-transfection, filtered through 0.45 μm membranes. Target cells were transduced with lentiviral particles at a multiplicity of infection (MOI) of 10 in the presence of 8 μg/mL polybrene (Sigma-Aldrich, St. Louis, MO, USA). Stable cell lines were selected using 2 μg/mL puromycin (Thermo Fisher Scientific) for 7 days.

### 2.9 Western blotting

Protein lysates were separated by sodium dodecyl sulfate-polyacrylamide gel electrophoresis (SDS-PAGE) and transferred onto polyvinylidene fluoride (PVDF) membranes (Millipore, Billerica, MA, USA). Membranes were blocked with 5% non-fat milk in Tris-buffered saline containing 0.1% Tween-20 (TBST) for 1 hour at room temperature, followed by overnight incubation at 4°C with the following primary antibodies diluted in TBST: anti-CD58 (1:500, Proteintech, Wuhan, China), anti-intercellular adhesion molecule-1 (ICAM-1, 1:1000, Proteintech), anti-PD-L1 (1:2000, Proteintech), anti-vimentin (1:2000, Proteintech), and anti-glyceraldehyde-3-phosphate dehydrogenase (GAPDH, 1:3000, Proteintech). After three washes with TBST, membranes were incubated with horseradish peroxidase (HRP)-conjugated secondary antibodies (1:3000, Zhongshan Golden Bridge Biotechnology, Beijing, China) for 2 hour at room temperature. Protein bands were visualized using an enhanced chemiluminescence (ECL) reagent (Advansta, Menlo Park, CA, USA) and imaged with a C-DiGit Blot Scanner (LI-COR Biosciences, Lincoln, NE, USA).

### 2.10 Wound healing assay

U87MG and LN229 cells were seeded in 6-well culture plates and grown to 90–100% confluence. A standardized scratch wound was generated in the cell monolayer using a sterile 200 μL micropipette tip. Cells were gently washed twice with phosphate-buffered saline (PBS; HyClone, Logan, UT, USA) to remove debris and incubated in serum-free DMEM. Cell migration into the wound area was monitored at 0, 24, and 48 hours using an inverted phase-contrast microscope (Nikon Eclipse Ts2, Tokyo, Japan). Images were analyzed with ImageJ software (version 1.53k; National Institutes of Health, Bethesda, MD, USA) by measuring the wound width at each time point. Three independent experiments were performed in triplicate.

### 2.11 Transwell assay

U87MG and LN229 cells (1 × 10^5^ cells) suspended in 100 μL serum-free DMEM were seeded into the upper chambers of 24-well Transwell plates (Corning Inc., Corning, NY, USA). The upper chambers were pre-coated with Matrigel basement membrane matrix (1 mg/mL; BD Biosciences, Franklin Lakes, NJ, USA) and fitted with polyethylene terephthalate membranes (8 μm pore size; Thermo Fisher Scientific, Waltham, MA, USA). The lower chambers were filled with DMEM supplemented with 10% FBS as a chemoattractant. After 48 hours of incubation at 37°C, non-invading cells on the upper membrane surface were removed using a cotton swab. Invaded cells on the lower surface were fixed with 100% methanol for 10 minutes, stained with 0.5% crystal violet (Sigma-Aldrich, St. Louis, MO, USA) for 20 minutes, and washed twice with PBS. Cell invasion was quantified by counting stained cells in five random fields per membrane under an inverted light microscope (Nikon Eclipse Ts2, 100× magnification; Nikon, Tokyo, Japan). Three independent experiments were performed in triplicate.

### 2.12 CCK8

U87MG and LN229 cells were seeded into 96-well culture plates at a density of 4 × 10³ cells per well (Corning Incorporated, Corning, NY, USA). After transfection with the indicated plasmids, cells were incubated in DMEM supplemented with 10% FBS for 24 hours. Cell viability was assessed using the Cell Counting Kit-8 (CCK-8; Beyotime Biotechnology, China) according to the manufacturer’s instructions. Briefly, 10 μL of CCK-8 reagent was added to each well containing 90 μL of serum-free DMEM, followed by incubation at 37°C for 2 hours. Absorbance was measured at 450 nm using a Multiskan Spectrum 1500 microplate reader (Thermo Fisher Scientific, Waltham, MA, USA). Three independent experiments were performed in triplicate.

### 2.13 Jurkat cell adhesion experiment

Control and CD58-knockdown U87MG/LN229 cells were seeded into 6-well plates at a density of 1 × 10^6^ cells per well and cultured to 80–90% confluence. Jurkat T cells (human acute T cell leukemia line; Chinese Academy of Medical Sciences, Beijing, China) (2 × 10^6^ cells/well) were co-cultured with glioma cells for 4 hours at 37°C. Non-adherent Jurkat cells were removed by three gentle washes with PBS (HyClone, Logan, UT, USA). Adherent Jurkat cells were visualized under an inverted fluorescence microscope (Nikon Eclipse Ts2, 20× objective; Nikon, Tokyo, Japan). Five random fields per well were imaged, and adherent Jurkat cells were manually counted and normalized to 100 glioma cells. Experiments were independently repeated three times with triplicate wells.

### 2.14 ELISA

U87MG and LN229 cells were seeded into 24-well culture plates at a density of 5 × 10^4^ cells per well and transfected with CD58 shRNA or control plasmids. After 48 hours of incubation, culture supernatants were collected and centrifuged at 1,000 × g for 10 minutes to remove cellular debris. Levels of CCL5, CXCL9, and CXCL10 were quantified using human ELISA kits (Elabscience Biotechnology Co., Ltd., Wuhan, China) according to the manufacturer’s instructions. Absorbance was measured at 450 nm using a Multiskan Spectrum 1500 microplate reader (Thermo Fisher Scientific, Waltham, MA, USA). Three independent experiments were performed in triplicate.

### 2.15 Statistical analysis

All data are presented as mean ± standard deviation (SD) and analyzed using SPSS software (version 23.0; IBM Corp., Armonk, NY, USA). Normality of data distribution was assessed by the Shapiro-Wilk test. Between-group differences were evaluated by one-way analysis of variance (ANOVA) with Tukey’s *post hoc* test for multiple comparisons or two-tailed Student’s t-test for pairwise comparisons, as appropriate. Categorical variables (e.g., immunohistochemical scores) were compared using the χ² test. Spearman’s rank correlation coefficient was applied to analyze associations between CD58 expression and clinicopathological parameters. Survival curves were generated by the Kaplan-Meier method, and statistical significance was determined by the log-rank test. A two-sided *p*-value < 0.05 was considered statistically significant. Three independent experiments were performed in triplicate unless otherwise specified.

## 3 Results

### 3.1 Pan-cancer expression profile and functional enrichment of CD58

**Figure 1:**
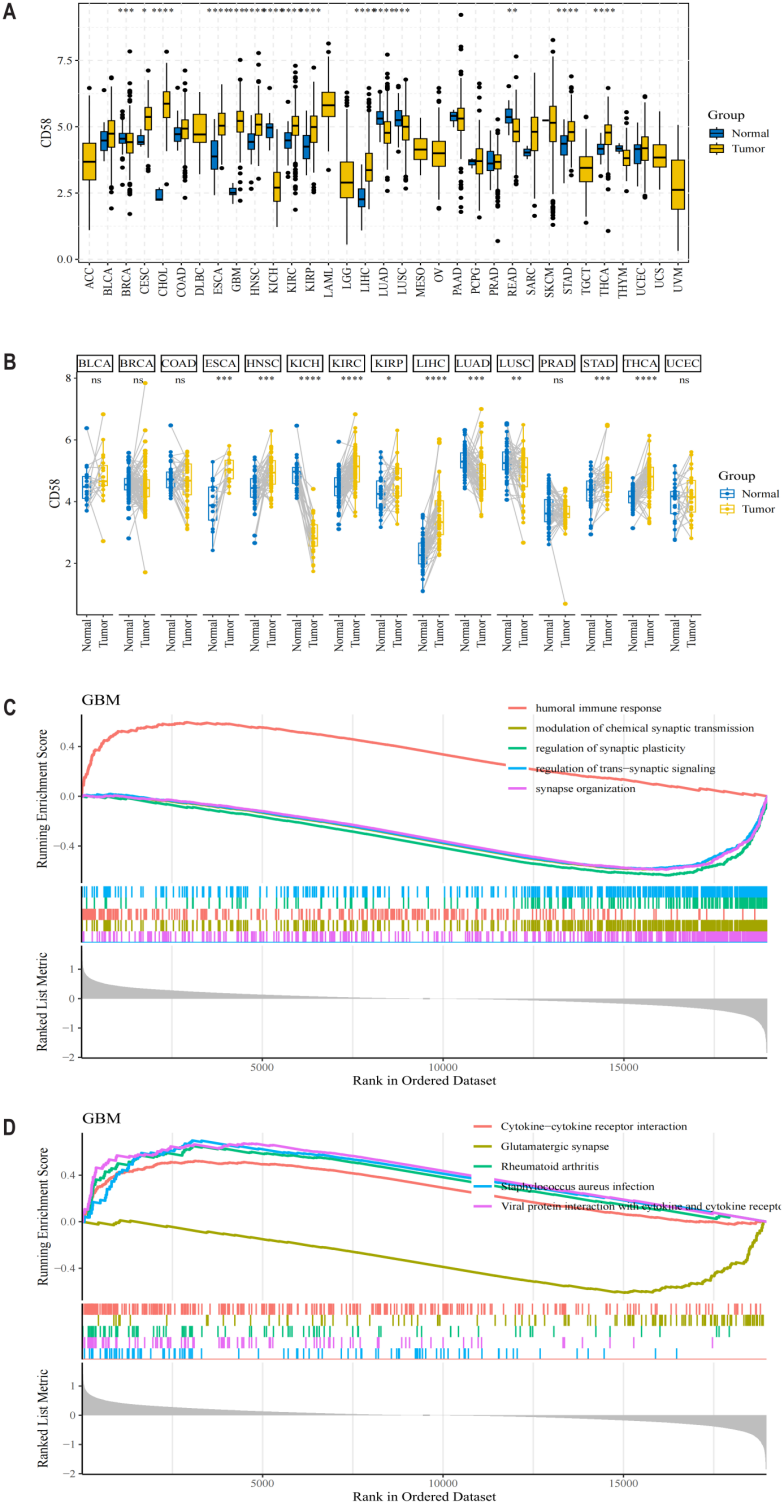
CD58 expression levels in different types of tumor tissues and normal tissues. **(A)** The mRNA expression level of CD58 in 33 types of cancer tissues and adjacent normal tissues from the TCGA database. Statistical significance was determined by the Wilcoxon rank-sum test (∗p < 0.05; ∗∗p < 0.01; ∗∗∗p < 0.001; ****p <0.0001). **(B)** The mRNA expression level of CD58 in paired tumor and normal tissues from the TCGA database. Statistical significance was determined by the Wilcoxon signed-rank test (∗p < 0.05; ∗∗p < 0.01; ∗∗∗p < 0.001; ****p <0.0001). **(C)** Gene Ontology (GO) enrichment analysis of genes correlated with CD58 expression. **(D)** Kyoto Encyclopedia of Genes and Genomes (KEGG) pathway enrichment analysis of genes correlated with CD58 expression.

To systematically characterize CD58’s role across malignancies, we analyzed its mRNA expression in 33 cancer types from TCGA database. CD58 was significantly upregulated in most tumors (**Figure 1A**), with notable overexpression in breast invasive carcinoma (BRCA), cervical squamous cell carcinoma (CESC), cholangiocarcinoma (CHOL), esophageal carcinoma (ESCA), glioblastoma (GBM), head and neck squamous cell carcinoma (HNSC), kidney chromophobe (KICH), kidney renal clear cell carcinoma (KIRC), kidney renal papillary cell carcinoma (KIRP), and liver hepatocellular carcinoma (LIHC) compared to adjacent normal tissues (*p* < 0.05). Conversely, CD58 expression was reduced in lung adenocarcinoma (LUAD) and lung squamous cell carcinoma (LUSC) (**Figure 1A**). Paired tumor-normal comparisons further confirmed CD58 upregulation in ESCA, HNSC, KICH, KIRC, KIRP, LIHC, LUAD, LUSC, stomach adenocarcinoma (STAD), and thyroid carcinoma (THCA) (**Figure 1B**). Functional enrichment analyses revealed CD58’s strong association with immune-related pathways: GO terms highlighted its involvement in immune response regulation (**Figure 1C**). The KEGG pathway analysis indicates that the interaction between cytokines and their receptor molecules might be one of the key mechanisms. (**Figure 1D**). After applying FDR correction, the GO and KEGG analyses revealed that CD58 was significantly enriched in immune-related pathways (such as cytokine-cytokine receptor interaction, FDR < 0.01).

### 3.2 CD58 correlates with immune checkpoint networks across cancers

**Figure 2:**
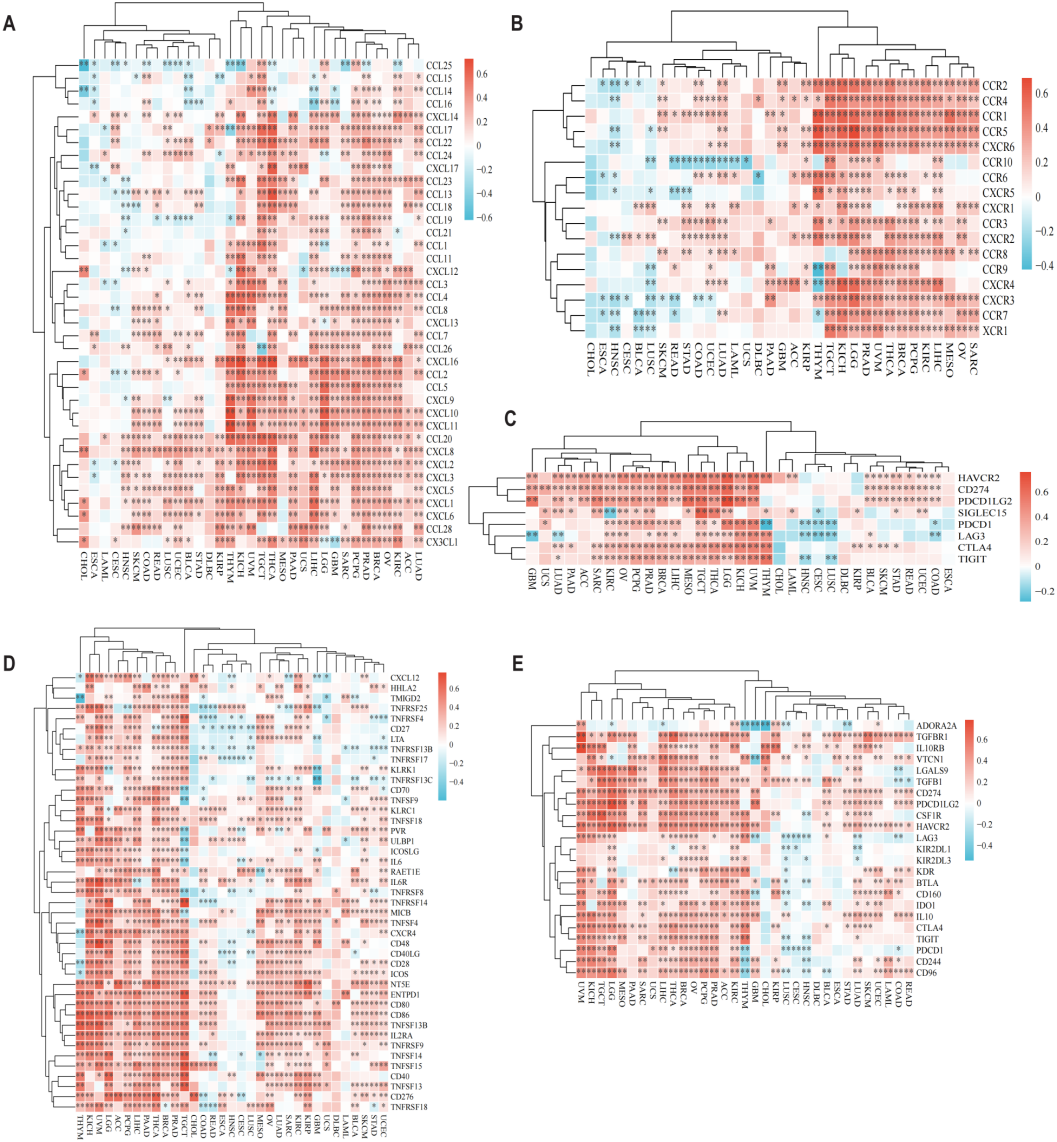
CD58 Pan-cancer immune function analysis and CD58 interacts with immune function and cytokines. **(A)** Heatmap of the correlation between chemokine and CD58 expression in pancancer. **(B)** Heatmap of the correlation between chemokine receptor and CD58 expression in pancancer. **(C)** Heatmap of the correlation between immune checkpoint genes and CD58 expression in pancancer. **(D)** Heatmap of the correlation between immune stimulation factor and CD58 expression in pancancer. **(E)** Heatmap of the correlation between immunosuppressants and CD58 expression in pancancer. (FDR-adjusted p-value Significant Codes: ∗p < 0.05; ∗∗p < 0.01; ∗∗∗p < 0.001).

To elucidate CD58’s immunoregulatory functions, we examined its associations with immune-related genes—including chemokines, chemokine receptors, immune checkpoints, immunostimulators, and immunosuppressors—across 33 cancer types in the TCGA dataset. Heatmap analysis demonstrated widespread correlations between CD58 and these immunomodulators in nearly all cancers, except for CHOL, ESCA, diffuse large B-cell lymphoma (DLBC), and uterine carcinosarcoma (UCS) (**Figures 2A–E**). In glioblastoma (GBM), the expression of CD58 is positively correlated with chemokines, C-C motif chemokine receptors, and immunosuppressive markers.

### 3.3 CD58 expression associates with immune cell infiltration in gliomas

**Figure 3:**
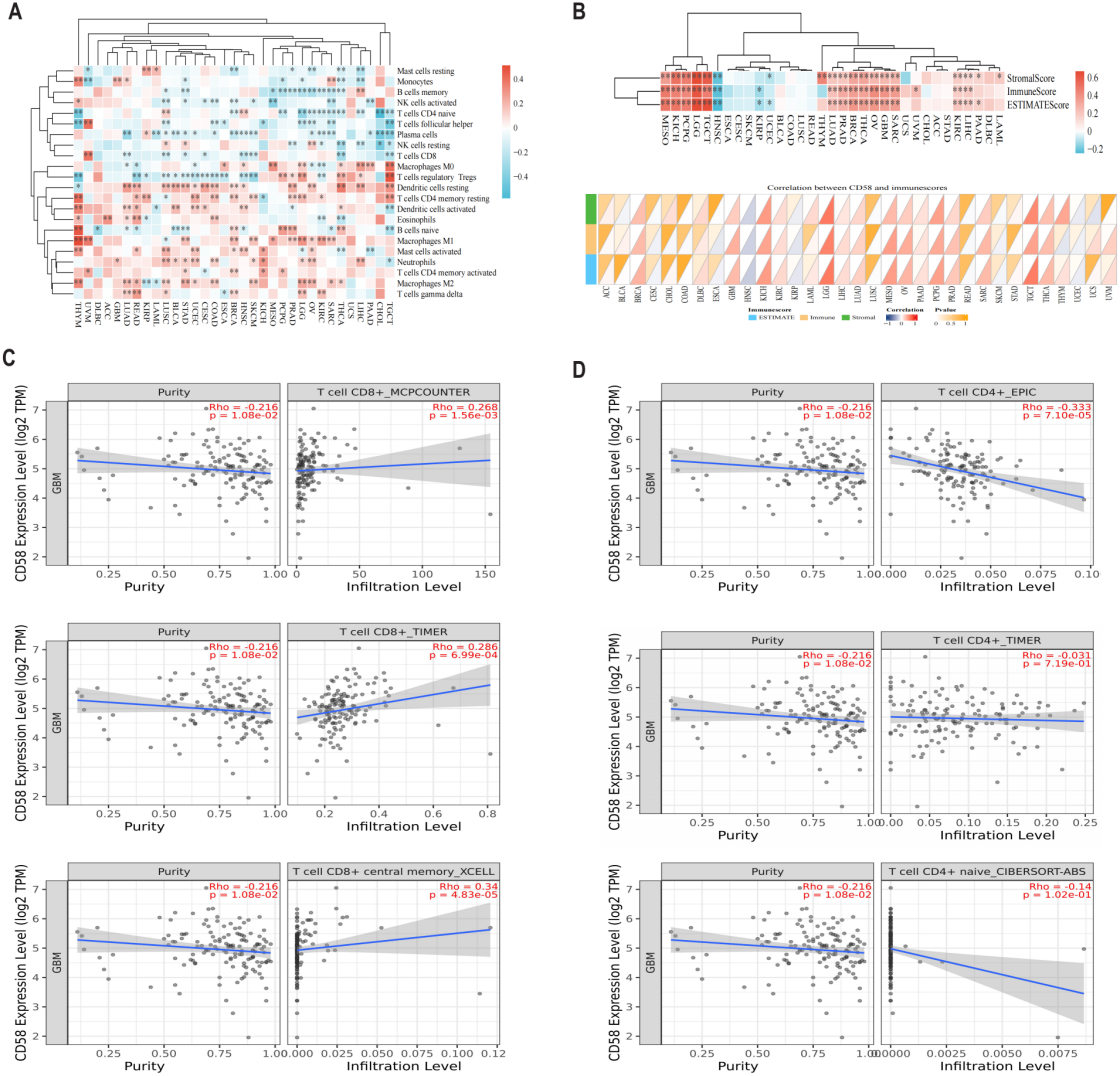
The relationship between CD58 and immune cell infiltration. **(A)** Bioinformatics analysis of the correlation between CD58 expression and immune effector cells using TISIDB database. **(B)** Heatmap of the correlation between immune score and CD58 expression in pancancer. **(C)** Correlation between CD58 expression and CD8+ T cell in GBM. **(D)** Correlation between CD58 expression and CD4+ T cell in GBM. (P value Significant Codes: ∗p < 0.05; ∗ ∗p < 0.01; ∗∗∗p <0.001).

To evaluate CD58’s impact on tumor immune contexture, we analyzed its correlation with immune cell infiltration levels in TCGA pan-cancer cohorts. CD58 expression showed significant positive associations with CD8^+^ T cells in 9 tumor types, regulatory T cells (Tregs) in 19 tumors, neutrophils in 12 tumors, and B cells in 11 tumors (**Figure 3A**). Furthermore, using the Estimation of Stromal and Immune cells in MAlignant Tumor tissues using Expression data (ESTIMATE) algorithm, we quantified TME components in GBM. CD58 expression strongly correlated with both immune scores (reflecting infiltrating immune cell abundance) and stromal scores (representing stromal cell proportions) (**Figure 3B**). Validation through multiple deconvolution algorithms—including Tumor Immune Estimation Resource (TIMER), Microenvironment Cell Populations-counter (MCPCOUNTER), xCELL, Estimation of Proportions of Immune and Cancer cells (EPIC), and Cell-type Identification By Estimating Relative Subsets Of RNA Transcripts (CIBERSORT)—confirmed CD58’s robust association with CD8^+^ T cell and CD4^+^ T cell infiltration in GBM (**Figures 3C, D**).

### 3.4 CD58 overexpression predicts poor prognosis in glioblastoma

**Figure 4:**
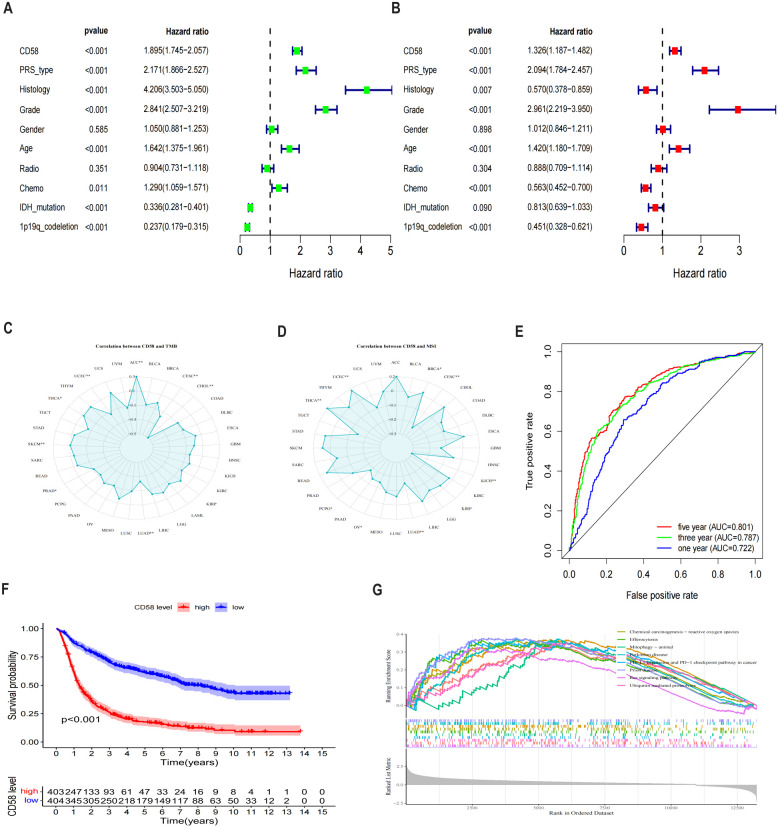
Analysis of prognostic factors of CD58 in GBM. **(A)** Forest plot of prognostic risk factors identified by univariate Cox regression analysis. HR, hazard ratio; CI, confidence interval. **(B)** Forest plot of independent prognostic risk factors identified by multivariate Cox regression analysis, adjusted for age, WHO grade, IDH status, and 1p/19q status. **(C)** Analysis of the Spearman correlation between CD58 expression and Tumor Mutational Burden (TMB) across various cancers (∗p < 0.05; ∗∗p < 0.01). **(D)** Analysis of the Spearman correlation between CD58 expression and Microsatellite Instability (MSI) across various cancers (∗p < 0.05; ∗∗p < 0.01). **(E)** Receiver Operating Characteristic (ROC) curves assessing the predictive accuracy of CD58 for 1-, 3-, and 5-year overall survival. AUC, area under the curve. **(F)** Kaplan-Meier survival curves comparing overall survival between GBM patients with high and low CD58 expression. Statistical significance was determined by the log-rank test. **(G)** Gene Set Enrichment Analysis (GSEA) plots showing signaling pathways enriched in the CD58 high-expression group. NES, normalized enrichment score; FDR, false discovery rate. (P value Significant Codes: ∗p < 0.05; ∗∗p < 0.01).

Univariate and multivariate Cox proportional hazards regression analyses identified high CD58 expression (hazard ratio [HR] = 1.895, 95% confidence interval [CI]: 1.745–2.057, *p* < 0.001) and advanced tumor stage (HR = 1.326, 95% CI: 1.187–1.482, *p* < 0.001) as independent prognostic risk factors (**Figures 4A, B**). To evaluate CD58’s potential in predicting immunotherapy response, we analyzed its correlations with tumor mutational burden (TMB) (**Figure 4C**) and microsatellite instability (MSI) (**Figure 4D**) across multiple cancers. CD58 expression showed significant associations with TMB in adrenocortical carcinoma (ACC), CESC, CHOL, LUAD, prostate adenocarcinoma (PRAD), skin cutaneous melanoma (SKCM), THCA, and uterine corpus endometrial carcinoma (UCEC). Similarly, CD58 correlated with MSI in breast invasive carcinoma (BRCA), CESC, KICH, LUAD, ovarian serous cystadenocarcinoma (OV), pheochromocytoma and paraganglioma (PCPG), THCA, and UCEC. The prognostic accuracy of CD58 was confirmed by ROC curves, with area under the curve (AUC) values of 0.722, 0.787, and 0.801 for 1-year, 3-year, and 5-year survival, respectively (**Figure 4E**). Kaplan-Meier survival analysis demonstrated significantly shorter OS in GBM patients with high CD58 expression compared to the low-expression group (*p* < 0.01; **Figure 4F**). GSEA further linked high CD58 expression to activation of the PD-L1 signaling pathway (**Figure 4G**).

### 3.5 Clinical and prognostic significance of CD58 expression in the CGGA cohort

**Figure 5:**
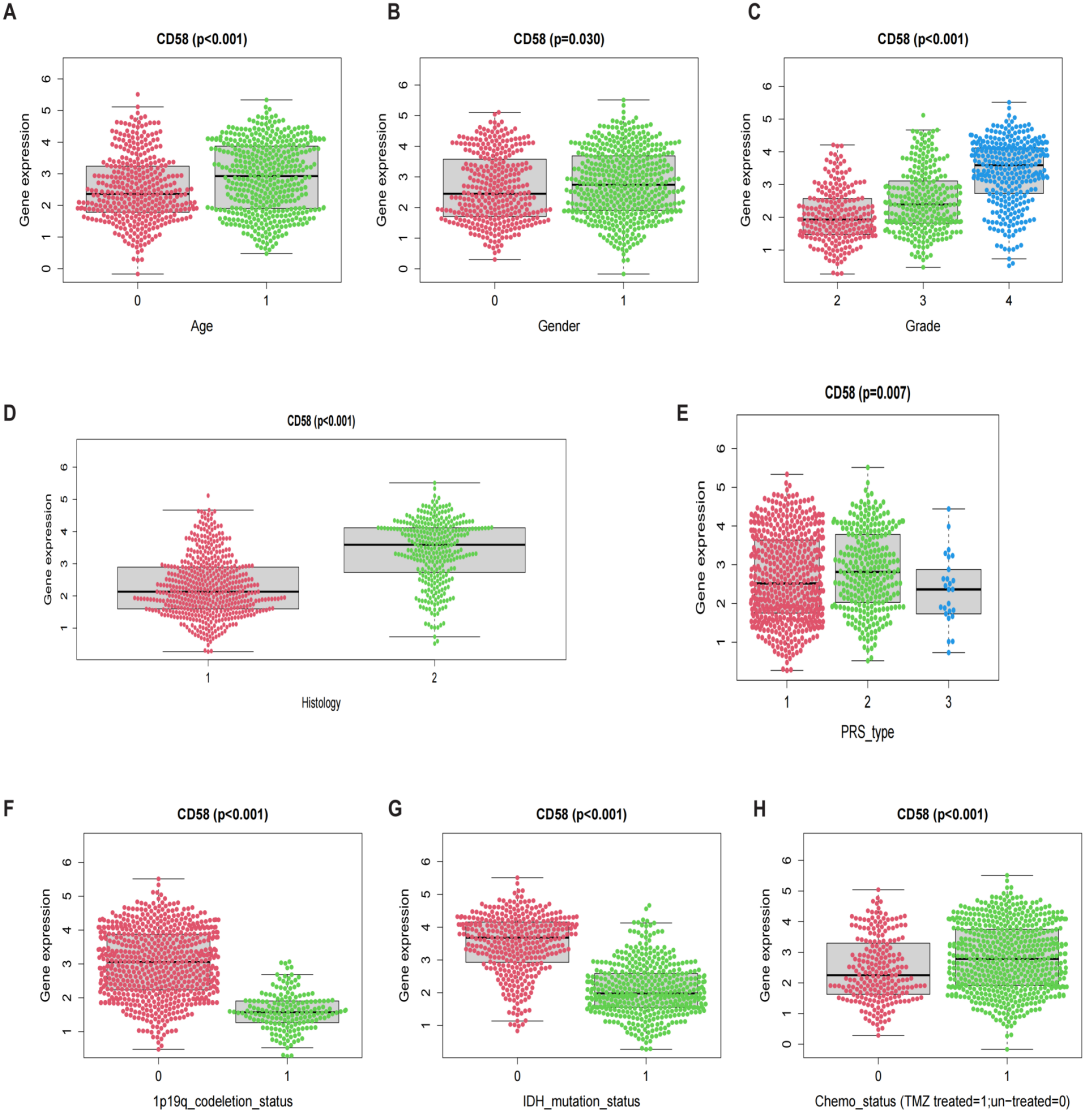
Prognostic factors: **(A)** Age. **(B)** Gender. **(C)** Grade. **(D)** Histology. **(E)** PRS. **(F)** 1p19q codeletion. **(G)** IDH mutation. **(H)** Chemo status.

To investigate the clinical relevance of CD58 in gliomas, we analyzed its expression across subgroups stratified by age, gender, WHO grade, histological subtype, progression-free survival (PRS) classification, 1p/19q codeletion status, IDH mutation status, and chemotherapy history, revealing significant associations with all evaluated parameters (*p* < 0.05; **Figures 5A–H**): CD58 expression was elevated in older patients (*p* < 0.001), male patients (*p* = 0.03), higher WHO grades (Grade II < III < IV; *p* < 0.001), IDH wild-type glioblastomas (*p* < 0.001), non-codeleted tumors (*p* < 0.001), and untreated cohorts (*p* < 0.001), while differing between PRS classifications (*p* < 0.001).

### 3.6 CD58 and PD-L1 staining intensity positively correlated with advanced tumor grade

**Figure 6:**
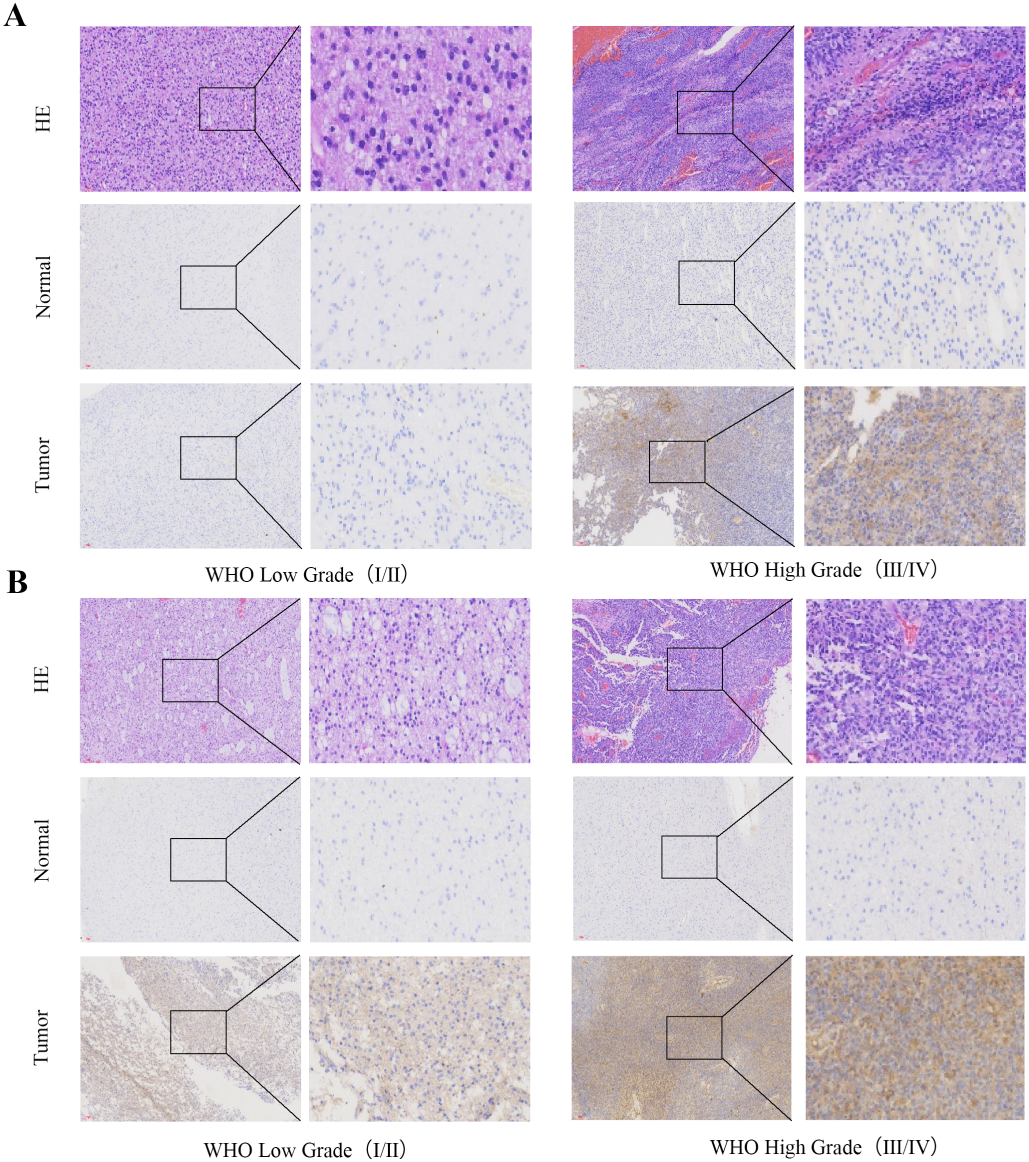
CD58 and PD-L1 staining intensity positively correlated with advanced tumor grade. **(A)** CD58 protein expression between tumor group and normal group in GBM (×200). **(B)** PD-L1 protein expression between tumor group and normal group in GBM (×200).

Immunohistochemical analysis demonstrated significantly higher expression of CD58 and PD-L1 in tumor tissues than in normal brain tissues. The staining intensity of CD58 and PD-L1 showed a positive correlation with advanced tumor grade (**Figures 6A, B**).

### 3.7 CD58 overexpression promoted metastasis of GBM cells

**Figure 7:**
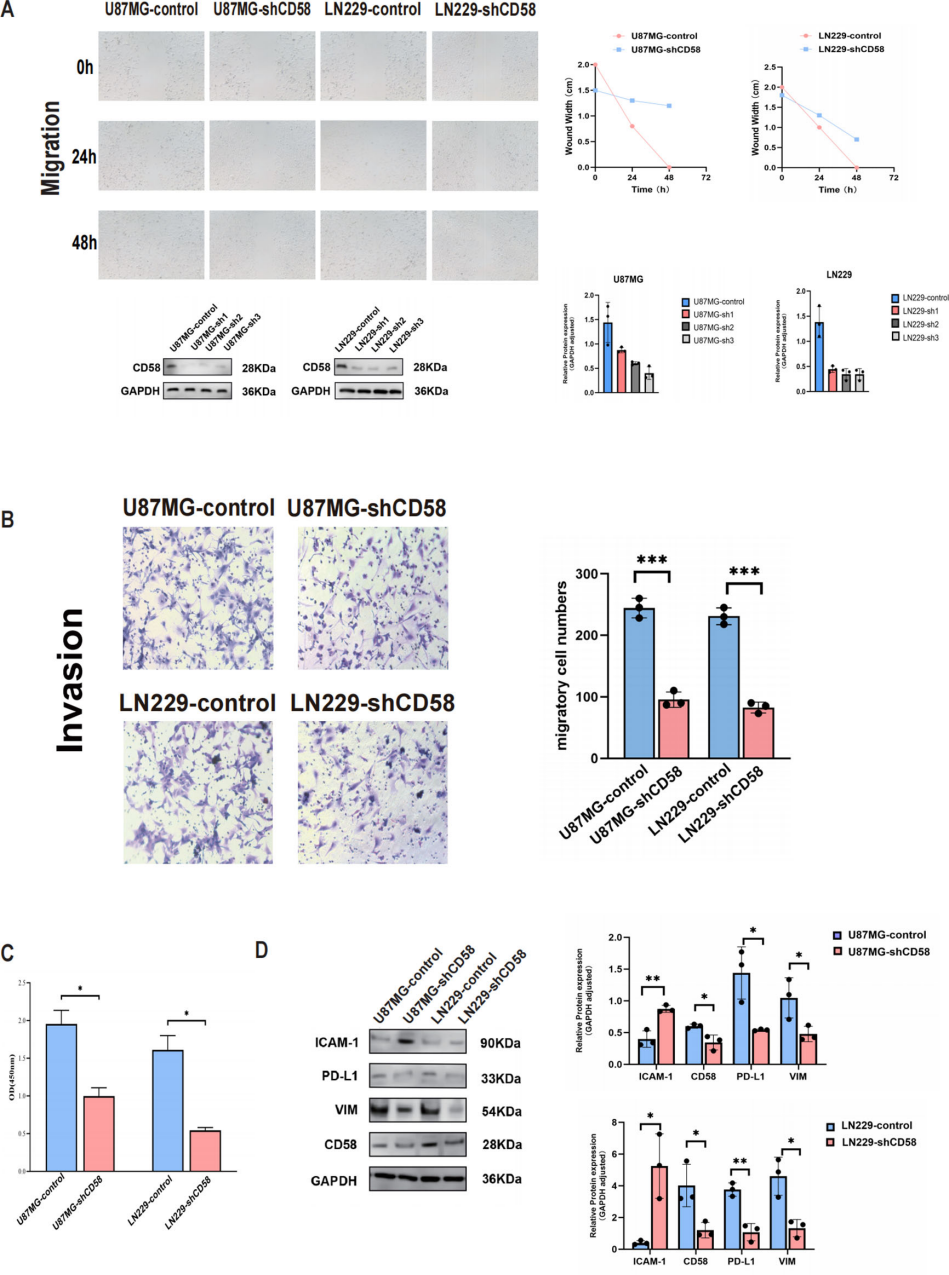
CD58 promoted GBM progression. **(A)** Wound healing assays evaluating the migration ability of control and CD58-knockdown U87MG and LN229 cells at 0, 24, and 48 hours. Quantification of the wound closure rate is shown (right). Data are presented as mean ± SD from three independent experiments (n=3). **(B)** Transwell invasion assays comparing the invasion ability of control and CD58-knockdown U87MG and LN229 cells. Representative images (left) and quantification of invaded cells (right) are shown. Data are presented as mean ± SD from three independent experiments, each performed with triplicate technical replicates (n=3). Statistical significance was determined by an unpaired Student’s t-test (***p < 0.001). **(C)** Cell Counting Kit-8 (CCK-8) assays detecting the viability of U87MG and LN229 cells after CD58 knockdown. Data are presented as mean ± SD from three independent experiments, each performed with triplicate technical replicates (n=3). (*p<0.05). **(D)** Western blot analysis of CD58, PD-L1, Vimentin, and ICAM-1 protein expression in control and CD58-knockdown U87MG and LN229 cells. GAPDH was used as a loading control. Blots are representative of three independent experiments (n=3). (P value Significant Codes: ∗p < 0.05; ∗∗p < 0.01).

To demonstrate the direct role of CD58 in GBM cells, U87MG and LN229 cells were transfected with a CD58 knockdown expression plasmid. Wound healing and transwell assays were conducted to assess the migration and invasion abilities of the GBM cells. *In vitro* wound healing was reduced in the GBM cells upon CD58 knockdown (**Figure 7A**). Blockade of CD58 also retarded invasion in U87MG and LN229 cells (**Figure 7B**). CCK-8 assays showed that CD58 silencing impaired the proliferation of GBM cells and enhanced their motility (**Figure 7C**). Next, the effect of CD58 on the expression of related proteins in glioblastoma cells was investigated. CD58 silencing reduced the expression of Vimentin. When CD58 was downregulated in U87MG and LN229 cells, the expression levels of the PD-L1 protein were significantly decreased. Furthermore, CD58 silencing increased the protein expression level of ICAM-1. Western blot analysis confirmed that CD58 silencing downregulated Vimentin and PD-L1 expression but upregulated ICAM-1 (**Figure 7D**).

### 3.8 CD58 modulates T cell adhesion and chemokine secretion in glioblastoma

**Figure 8:**
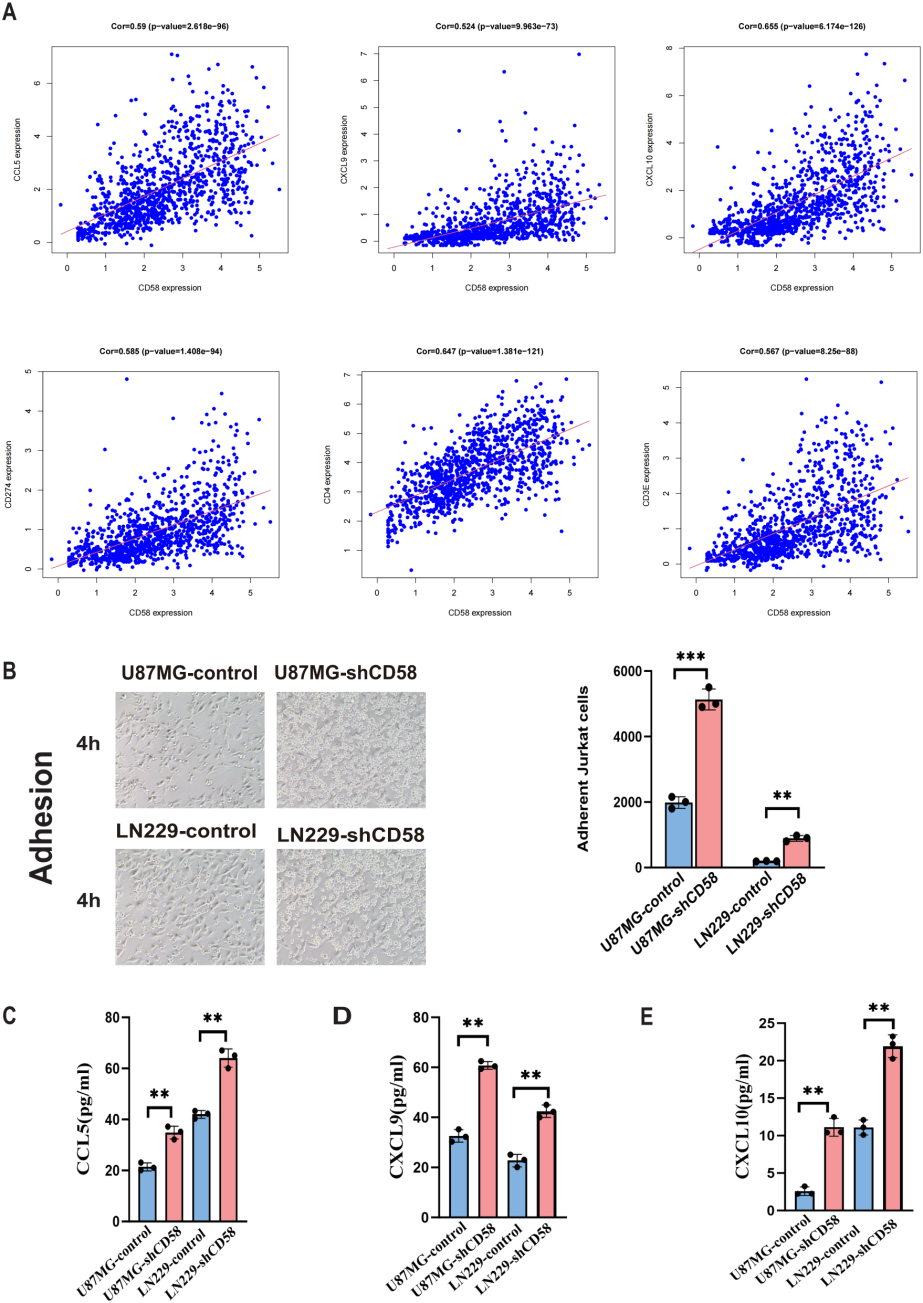
Correlation analysis of target gene CD58 and immune genes. **(A)** Correlation scatter plots of CD58 expression with immune-related genes (CCL5, CXCL9, CXCL10, CD274/PD-L1, CD4, CD3) in GBM. Pearson correlation coefficients (Cor) and p-values are indicated. **(B)** Jurkat T cell adhesion assays comparing the adhesion ability of control and CD58-knockdown U87MG and LN229 cells. The number of adherent Jurkat cells per 100 glioma cells was quantified. Data are presented as mean ± SEM from three independent experiments, each performed with triplicate technical replicates (n=3). Statistical significance was determined by an unpaired Student’s t-test (***p < 0.001). **(C–E)** ELISA quantification of the secretion levels of chemokines (CCL5, CXCL9, CXCL10) in the conditioned media from control and CD58-knockdown U87MG and LN229 cells. Data are presented as mean ± SD from three independent experiments, each performed with triplicate technical replicates (n=3). Statistical significance was determined by an unpaired Student’s t-test (**p < 0.01).

Highly expressed CD58 correlates with an immunosuppressive tumor microenvironment. To study the regulatory mechanism of CD58 in the tumor microenvironment, we further analyzed the associations of CD58 with chemokines and PD-L1, immune-related genes, and found that the expression of CD58 was correlated with most chemokines. After applying FDR correction(FDR < 0.01), CD58 was compared with CCL5 (Cor=0.59, p=2.618e-96), CXCL9 (Cor=0.524, p=9.963e-73), and CXCL10 (Cor=0.655, p= 6.174e-126), CD274 (Cor=0.585, p=1.408e-94), CD4 (Cor=0.647, p=1.381e-121), CD3 (Cor=0.567, p=8.25e-88) (**Figure 8A**). Combining these statistical data, we conducted relevant verifications by integrating cell adhesion experiments and ELISA detections.

The adhesion experiment assesses the influence of GBM cells on T cell adhesion. T cell adhesion significantly increased in GBM cells with low CD58 expression compared to the control group (**Figure 8B**). To elucidate the changes in T cell adhesion, we conducted ELISA assays to measure chemokine secretion in the cell culture media of different groups. Results indicated that reduced CD58 expression led to a significant increase in CCL5, CXCL9, and CXCL10 secretion compared to the control (**Figures 8C–E**).

## 4 Discussion

Glioma, a highly aggressive malignancy in the central nervous system, is known for its immunosuppressive tumor microenvironment (TME) and resistance to standard treatments, presenting substantial clinical hurdles (1, 13). Our integrated analysis nominates CD58 as a potential independent prognostic biomarker and a crucial modulator of immune evasion in glioma. Our pan-cancer analysis reveals its consistent upregulation in 33 malignancies, including GBM, where it strongly correlates with immunosuppressive molecules (PD-L1, CTLA-4, TGF-β, IL-10), suggesting a potential universal role in immune evasion. The CD58-CD2 axis is pivotal for productive immune synapse formation and T/NK cell activation. Notably, CD58 loss—rather than CD2 deficiency—is a primary mechanism of axis disruption, leading to impaired T-cell cytotoxicity and tumor immune evasion (11, 14). Additionally, CD58 competes with PD-L1 for binding to chemokine-like factor 6 (CMTM6), a key regulator of lysosomal degradation. Loss of CD58 redirects CMTM6 to stabilize PD-L1, exacerbating immunosuppression (15). High CD58 expression in diffuse large B cell lymphoma correlates positively with increased immune cell presence, while reducing CD8+ T cell depletion (16). Wu et al. have associated CD58 with tumor progression and poor prognosis in glioma patients (12). Prior research has linked activated CD58 with tumor promotion in colorectal cancer and hepatocellular carcinoma through upregulation of the Wnt/β-catenin pathway (17, 18). In pancreatic cancer, elevated CD58 levels are linked to unfavorable outcomes, vascular invasion, and metastasis (19). These collective findings underscore the multifaceted and intricate role of CD58 in tumor immunity. Notably, in this investigation, CD58 expression significantly surpassed levels in low-grade gliomas (WHO I-II) in high-grade gliomas (WHO III-IV), particularly in aggressive molecular subtypes like IDH wild-type and 1p19q non-codeletion. Our analysis revealed a significant disparity between CD58 expression and T cell infiltration in gliomas. The notable association of CD58 with IDH wild-type and 1p19q non-co-deletion gliomas implies a relatively “cold” immune microenvironment in IDH mutant gliomas characterized by low T cell infiltration (20, 21). Conversely, heightened CD58 expression in IDH wild-type tumors may indicate a greater reliance on the PD-L1 pathway, rendering them suitable for targeted therapeutic interventions. Oligodendrogliomas lacking 1p19q co-deletion exhibit sensitivity to chemotherapy but are predisposed to recurrence, suggesting that CD58-targeted therapy could potentially delay disease recurrence. Patients exhibiting high CD58 expression demonstrated significantly shorter median survival times compared to those with low CD58 expression. Both univariate and multivariate Cox regression analyses confirmed the independence of CD58 expression from conventional prognostic factors such as age and WHO classification. These findings underscore CD58 overexpression as a prognostic risk factor for gliomas, suggesting its potential utility as a supplementary marker for glioma prognosis and molecular stratification.

Our research demonstrates that CD58 promotes glioma cell proliferation, invasion, and migration, consistent with previous findings showing elevated CD58 levels correlate with poor prognosis in various tumors (17–19, 22). Notably, heightened CD58 expression in gliomas leads to a notable increase in macrophage infiltration, which exhibit immunosuppressive properties, aiding tumors in evading immune surveillance and immune effector cell attacks (23). Conversely, CD58 expression is associated with a decrease in CD8+ T cell infiltration in glioma tissues (12), consistent with our own experimental outcomes. Suppression of CD58 has been found to stimulate the release of T cell-related chemokines and enhance adhesion molecule expression on tumor cell surfaces, indicating CD58’s role in reshaping the tumor immune microenvironment. In IDH wild-type gliomas, a substantial accumulation of myeloid-derived suppressor cells (MDSCs) occurs. These MDSCs facilitate tumor cell proliferation through growth factor secretion and impede T cell function to enable tumor immune evasion, ultimately establishing a positive feedback loop that propels tumor advancement (24). In this investigation, we demonstrated a significant reduction in PD-L1 protein levels in glioma upon CD58 down-regulation, as evidenced by functional enrichment analysis and Western blotting. Mechanistically, the transcription of PD-L1 may be facilitated by CD58-mediated activation of STAT3 signaling, as previous studies have indicated that activated STAT3 protein enhances PD-L1 transcriptional expression (25). Wu et al. reported elevated levels of IL-6, IL-8, and IL-10 in CD58-overexpressing glioma cells, with IL-6 stimulating PD-L1 expression through STAT3 and NF-κB pathways (12, 26), aligning with our observations. Our investigation revealed a positive correlation between CD58 expression and the immunosuppressive marker PD-L1, as well as with CD8+ T and CD4+ T cells. Furthermore, we observed CD58 expression across various cell culture groups, influencing the secretion of chemokines CCL5, CXCL9, and CXCL10. CCR5 facilitates T cell migration to inflammatory or infectious sites by binding to chemokines like CCL3, CCL4, and CCL5, particularly crucial in anti-tumor immunity and chronic inflammation, the infiltration of CCR5+ T cells is associated with the efficacy of immunotherapy. The CCL5/CCR5 axis enhances T cell migration to the tumor site (27). CXCL9 and CXCL10 are linked to T cell infiltration in various solid tumors (28). These chemokines are induced by IFN-γ, recruit CD8+ T cells, and correlate positively with immunotherapy response (29). The upregulation of PD-L1 in CD58-overexpressing tumors is consistent, affecting T cell infiltration and reshaping the immune microenvironment. This dysregulation of cytokine levels may lead to the development of an immune desert microenvironment and the inhibition of effector T cell activity. Notably, experiments on cell adhesion revealed that CD58 cells with low expression enhanced their capacity to bind to T cells. This finding suggests that CD58 cells may facilitate interactions between tumor cells and immune cells by modulating the expression of the adhesion molecule ICAM-1 on tumor cell surfaces, thereby influencing the immune microenvironment.

The PD-L1/PD-1 axis-based therapeutic regimen has demonstrated efficacy in various solid tumors, but its effectiveness remains limited in gliomas (30, 31). Our research reveals a persistent elevation of PD-L1 in gliomas overexpressing CD58, leading to T cell depletion and the creation of an immunosuppressive microenvironment that hinders effector T cell function, resulting in an “immune desert.” Targeting CD58 with monoclonal antibodies shows promise in reversing T cell suppression and augmenting the effects of PD-1 blockade. This investigation highlights CD58 as a potential crucial target for overcoming resistance in gliomas, with implications for different prognostic groups: individuals exhibiting high CD58 expression may derive early therapeutic benefits from combination therapies involving PD-1 inhibitors.

This study has limitations. The reliance on knockdown approaches without complementary rescue experiments means we have established the necessity but not the sufficiency of CD58 for the observed phenotypes. Furthermore, the absence of *in vivo* validation limits our understanding of CD58’s function within the complex tumor microenvironment. Future studies incorporating rescue assays and animal models are warranted to fully elucidate the role of the CD58-PD-L1 axis.

## 5 Conclusions

This study systematically elucidates the molecular mechanisms through which CD58 enhances glioma progression by increasing PD-L1 expression and reshaping the immunosuppressive microenvironment. These findings underscore CD58’s dual significance as both a prognostic biomarker and a therapeutic target in glioma. Targeting the CD58-PD-L1 axis presents a novel approach to overcoming the challenges in glioma immunotherapy. However, additional investigations into the mechanisms involved and translational research are imperative to advance its clinical implementation.

## Data Availability

The original contributions presented in the study are included in the article/supplementary material. Further inquiries can be directed to the corresponding authors.
